# Integrating High Resolution Water Footprint and GIS for Promoting Water Efficiency in the Agricultural Sector: A Case Study of Plantation Crops in the Jordan Valley

**DOI:** 10.3389/fpls.2016.01877

**Published:** 2016-12-14

**Authors:** Eliav Shtull-Trauring, Ido Aviani, Dror Avisar, Nirit Bernstein

**Affiliations:** ^1^Institute of Soil Water and Environmental Sciences, ARO, The Volcani CenterBet-Dagan, Israel; ^2^The Water Research Center, School of Earth Sciences, Faculty of Exact Sciences, Tel Aviv UniversityTel Aviv, Israel; ^3^EcoPeace Middle-EastTel Aviv, Israel

**Keywords:** agriculture, gray water, banana, palm dates, avocado, environment, water pollution, drainage basin

## Abstract

Addressing the global challenges to water security requires a better understanding of humanity's use of water, especially the agricultural sector that accounts for 70% of global withdrawals. This study combined high resolution-data with a GIS system to analyze the impact of agricultural practices, crop type, and spatial factors such as drainage basins, climate, and soil type on the Water Footprint (WF) of agricultural crops. The area of the study, the northern Lower Jordan Valley, covers 1121 ha in which three main plantation crops are grown: banana (cultivated in open-fields or net-houses), avocado and palm-dates. High-resolution data sources included GIS layers of the cultivated crops and a drainage pipe-system installed in the study area; meteorological data (2000–2013); and crop parameters (yield and irrigation recommendations). First, the study compared the WF of the different crops on the basis of yield and energy produced as well as a comparison to global values and local irrigation recommendations. The results showed that net-house banana has the lowest WF based on all different criteria. However, while palm-dates showed the highest WF for the yield criteria, it had the second lowest WF for energy produced, emphasizing the importance of using multiple parameters for low and high yield crop comparisons. Next, the regional WF of each drainage basin in the study area was calculated, demonstrating the strong influence of the Gray WF, an indication of the amount of freshwater required for pollution assimilation. Finally, the benefits of integrating GIS and WF were demonstrated by computing the effect of adopting net-house cultivation throughout the area of study with a result a reduction of 1.3 MCM irrigation water per year. Integrating the WF methodology and local high-resolution data using GIS can therefore promote and help quantify the benefits of adopting site-appropriate crops and agricultural practices that lower the WF by increasing yield, reducing water consumption, and minimizing negative environmental impacts.

## Introduction

Water is the ultimate renewable resource, yet in many areas of the world, especially in arid and semi-arid area, surface (lakes and reservoirs) and sub-surface (aquifers) water resources are at record-low levels and suffer of increasing levels of pollution. Climate change, spread of urbanization, and large-scale water diversion projects will further reduce the availability of water, especially in arid and semi-arid regions, while population growth and improvements in the standard of living will increase demand. The expected combined effect of these factors is the intensification of existing pressures on water-resources throughout the twenty-first century (Vörösmarty et al., 2010; Turral et al., 2011; Richey et al., 2015). Failure to address these pressures can lead to adverse changes in water quality that will affect human health, ecosystems, and water availability (Field et al., 2014).

Agriculture, the largest global water consumer, accounts for up to 70% of global withdrawals (Calzadilla et al., 2010; FAO, 2015). Furthermore, the food demand is expected to double by 2050, far above the expected crop production growth rate (Rak et al., 2013). Therefore, proper management, utilization and understanding of water consumption in the agricultural sector is key in tackling the growing threats of water shortages and the resulting geopolitical instability caused by increasing food prices (Molden, 2007).

The concept of virtual water, i.e., the amount of water consumed in the process of producing each product, was developed as a framework describing the global water trade (Allan, 2003). Each product that is shipped across the globe can be expressed by the volume of virtual water utilized for its production. In arid/semi-arid regions virtual water import is an important tool for reducing pressures on local water resources (Allan, 1997; Hakimian, 2003). However, even in countries that rely heavily on virtual water imports such as Egypt, Jordan, Libya, and Israel, agriculture is a major water consumer (Hoekstra and Chapagain, 2007; Hoekstra and Mekonnen, 2012; IWA, 2012; FAO, 2015). Understanding and mapping a country's water-use is an important step toward increasing water-use efficiency and water security (Molden et al., 2003). The Water Footprint (WF) methodology was developed for this purpose, expanding on the concept of virtual water to create a quantitative tool of water consumption globally, of a specific country, drainage basin, industry, business, product, service, or individual (Hoekstra et al., 2011).

The WF of an agricultural crop comprises of the evapotranspiration (ET) of irrigation water (WF_blue_) and rain water (WF_green_), most of which is consumed via crop transpiration (Allen et al., 1998; Hoekstra et al., 2011). The WF_green_ of agriculture is not accounted today as part of the crop water requirement in many countries. A better understanding and accounting of crop WF_green_ is essential for encouraging wide-spread utilization of rain-management practices. These practices, for example improved tillage and mulching practices, can increase the productive green-water consumption, i.e., the rain water available for crop transpiration, while reducing potential soil-erosion and flooding damages caused by storm-water run-off (Falkenmark and Rockström, 2006; Rockström et al., 2009). A third component of WF, the WF_gray_, is the volume of water necessary for diluting byproduct pollutants (such as fertilizers and pesticides) that reach ground or surface water resources. WF_gray_ is the volume of water required to dilute the chemical substance so its concentration in the receiving water body remains below the accepted water quality standard (Franke et al., 2013). The WF is expressed as volume (m^3^) per unit of product (usually as yield—ton^−1^ in fresh weight). Therefore, WF_blue_ and WF_green_ serve as an indicator of the water use efficiency (WUE) of crops. This normalization allows to compare crops grown under irrigation with different water qualities, climatic conditions and growth practices. WF can also be expressed as volume (m^3^) per unit of energy produced, or profit earned, that provide additional indicators of water efficiency (Hoekstra et al., 2011).

WF studies for crops were conducted with a global (Mekonnen and Hoekstra, 2011), country (Aldaya et al., 2010; Ge et al., 2011), or drainage basin (Mekonnen et al., 2012; Dumont et al., 2013) scope. One limiting factor of these studies is the use of global database inputs of climate factors for ET calculations with a resolution of 5 by 5 arc minutes. This allows analysis for large areas at a relatively low spatial resolution, thereby limiting the accuracy of the results (Hoekstra et al., 2011). Increasing the spatial resolution and analyzing the spatial factors influencing the WF can be facilitated through the use of a Geographic Information System (GIS).

Integrating GIS with WF can provide a number of benefits. Foremost, the effects of spatial factors such as climatic conditions or soil type, and temporal factors such as seasonal and yearly changes in climatic conditions, can be evaluated with high resolution using GIS. Additionally, GIS allows to easily calculate the WF of the total area studied (regional WF) by multiplying the WF_crop_ by the aggregate field sizes in the region of study. Furthermore, beyond describing the existing WF status, GIS allows modeling the impact of changes in agricultural practices and spatial distributions of different crops. This feature can help in cost-benefit analysis of the implementing practices that reduce water consumption and increase efficiency (Fortes et al., 2005; Liu, 2009; Thorp et al., 2015; Singh, 2016). Thus, the use of GIS technology using high-resolution data can provide spatial and temporal advantages that facilitate a better understanding of the WF of the current or alternative situations in the studied area. GIS provides different tools for the spatial analysis of the WF results and the ability to display these results on maps that can help clarify the impact of the different spatial aspects on the WF.

There have been a few attempts to combine WF with GIS technology. One study checked the WF of different river-basins in Greece on the basis of estimating WF of distinct land-uses (Ines et al., 2002), even software and models developed toward this aim (Fortes et al., 2005; Liu, 2009; Multsch et al., 2011, 2013). The topic of this paper, a WF study in the Jordan Valley is the first time a WF study was produced based on local-scale, high spatial resolution (field level) data. This was made possible by a GIS vector-layer that included data regarding crop type, cultivation practice, irrigation water source and quality, and planting date on a spatial resolution of 0.1 ha. Data from this map was used to compare the WF of three crops—Banana, Avocado, and Palm-Dates, the main plantation crops in the study area. The banana crop was divided into two sub-categories—cultivation in an open field (open-field banana) and in net-houses (net-house banana), and were treated as two distinct cropping systems. Over the past decade around half of the area devoted to growing bananas in the study area was covered with net-houses. This practice has shown benefits including reducing the required irrigation water volume as well as increasing the crop yield (Israeli et al., 2009).

A unique feature of the studied area, contributing to its suitability as a model area for WF studies of agricultural crops, is the existence of a large scale underground drainage system. The drainage system was installed in the 1960s when flood irrigation was still commonplace in the region. This extensive system of pipes, installed 1.5–2 m underground, spans a total length of ~112 km over an area of 2470 ha. The pipes collect and drain agricultural leachates from the different cultivated fields into the Sea of Galilee, Jordan River, and Yarmouk River. A GIS layer of the drainage pipe system installed under the studied area allowed to delineate the different drainage basins—necessary for the WF_gray_ calculations.

The WF_gray_ calculations were based on the water requirements to neutralize nitrogen (N) and phosphorous (P) pollutions through their use as agricultural inputs for the different crops. This decision was based on the understanding that fertilizers, despite their importance for plant development and yield increase, are also a main global source of water pollution (Carpenter et al., 1998; Rockström et al., 2009; Good and Beatty, 2011). In the area of study, changes in nutrient loading to the sea of Galilee that increased P uptake and altered N:P ratio, resulted in conditions that favor cyanobacteria dominance (Gophen et al., 1999). Agricultural runoff also resulted in high levels of N in the Lower Jordan River (Segal-Rozenhaimer et al., 2004). Despite the relatively high concentrations of N and P in the water sources in the area of study, there is no ongoing monitoring of the contribution of agricultural runoff to nutrient loading to the water sources. This provided a strong motivation for performing a WF_gray_ based on nutrient pollution, calculated using the methodology outlined by Franke et al. (2013).

The main aim of this study was to use the WF study in the Jordan Valley area as a test case that will allow to analyze the benefits of integrating WF with GIS based on high resolution data. In areas, such as Israel, where high spatial resolution agricultural, climate, and soil data is available, this integration can provide information that can be used by policy makers, farmers and researchers to evaluate and better understand the impact of implementing agricultural practices that increase water efficiency (WF_blue_ and WF_green_) and minimize environmental damage (WF_gray_).

## Materials and methods

**Figure 1:**
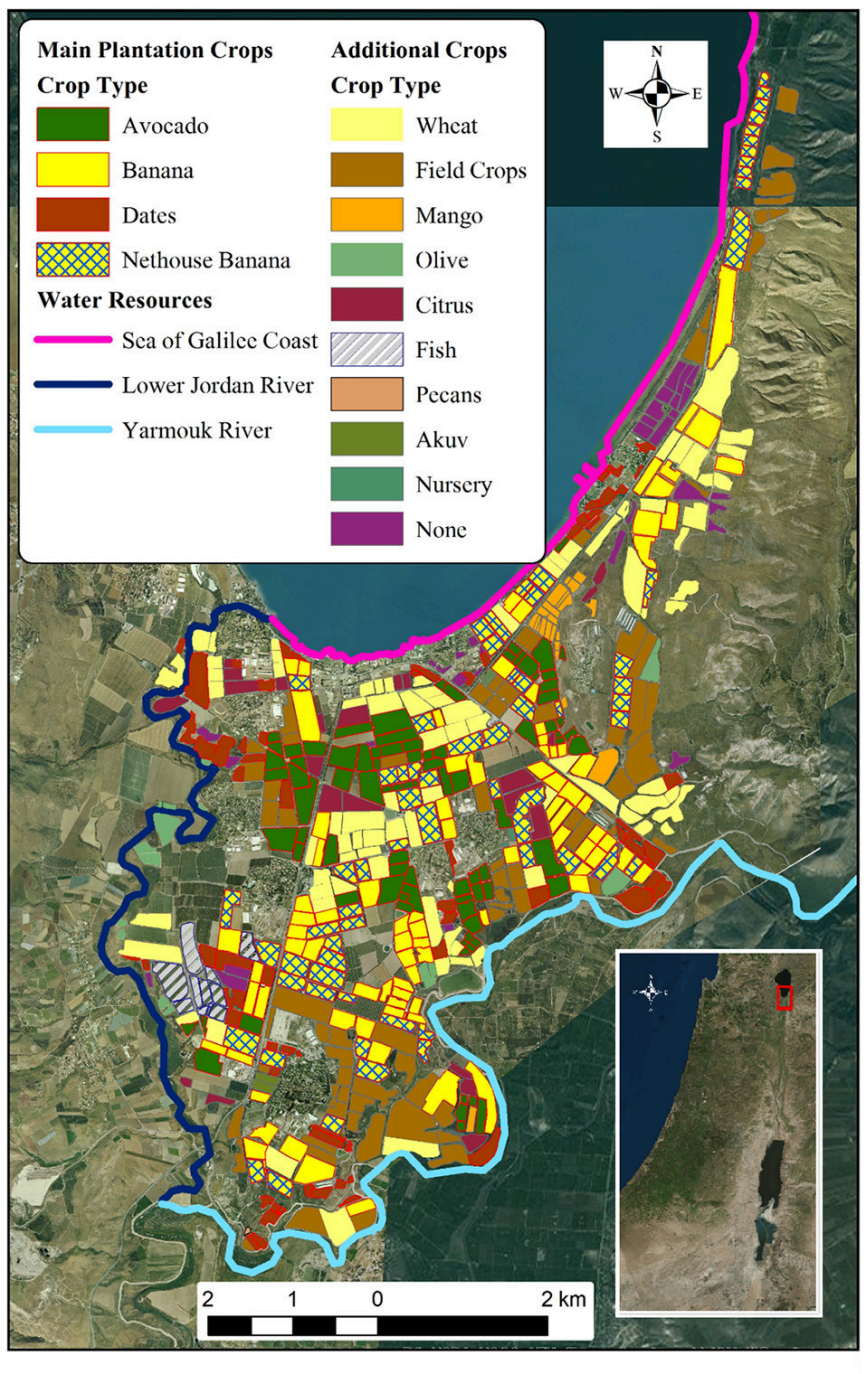
**Distribution of crops and main water resources in the area of study (source: MOAG)**.

The study was performed in the area of *Kikar Ha'Yarden*, the northern end of the Lower Jordan Valley, Israel (see Figure 1). The area can be described as a triangle comprised of the three edges of the Lower Jordan River, the *Yarmouk* River [YR] and the southern coast of the Sea of Galilee; and its three vertices at *Naharayim*, where the two rivers meet, the Lower Jordan River source at *Degania* Bridge and the northern end of *Kibuttz Ha'on*'s banana fields. The area is mostly used as an agricultural land cultivated by 10 farming communities (Kibbutzim).

Data regarding crop distribution and field size was based upon a GIS map in a resolution of 0.1 hectare provided by the Israeli Ministry of Agriculture based on data from 2012. The total documented area (<~3–5% of the area were not included in the map) is 2141.4 hectares [ha]: more than half (1280.6 ha) are plantation crops, 397.4 ha of wheat, 390.1 ha uncultivated land, 47 ha are fish ponds and 8.2 ha of other crops (Figure 1). The four main crops chosen for this study are open-field banana net-house banana, avocado, and palm-dates. These four crops constitute almost 90% (1121 ha) of the plantation crops grown in the area.

### WF_crop_: WF_blue_, WF_green_, and WF_gray_ calculation

The WF analysis for the crops followed the guidelines provided in the WF Assessment Guide [WFAM] (Hoekstra et al., 2011) and was calculated based on Equation (1):

(1) $$\begin{array}{l} \text{W}{\text{F}}_{\text{crop}}=\text{W}{\text{F}}_{\text{blue}}+\text{W}{\text{F}}_{\text{green}}+\text{W}{\text{F}}_{\text{greay}}\;\left[\text{volume mas}{\text{s}}^{-1}\right] \end{array}$$

where WF_blue_ represents consumption of irrigation water; WF_green_ represents consumption rain water; and WF_greay_ represents the total amount of water required to dilute the pollutants so their concentration remains below the accepted standard of the receiving water body. Together they comprise the WF_crop_, the total amount of water consumed during the process of growing a crop, i.e., no longer available for alternative uses.

The water consumption of a crop for WF_blue_ and WF_green_ is determined by the crop evapotranspiration (ET), i.e., the amount of water transpired by a crop and evaporated from the soil. During a crop's life cycle, the vast majority of water is lost via transpiration, e.g., over 90% for a crop with fully developed canopy cover (Hoekstra et al., 2011).

(2) $${\text{WF}}_{\text{blue}}=\frac{\text{CWU}.\text{blue}}{\text{Y}}\;[\text{volume}\;{\text{mass}}^{-1}]$$

(3) $${\text{WF}}_{\text{green}}=\frac{\text{CWU}.\text{green}}{\text{Y}}\;[\text{volume}\;{\text{mass}}^{-1}]$$

where CWU is crop water use, the volume of water (m^3^) used by crop for evapotranspiration and no longer available for other uses. CWU._blue_ is derived from surface or underground sources—or irrigation water, and CWU._green_ derived from rain water. CWU._blue_ + CWU._green_ is the ET from a field of a determined size (ha) during a defined time period (year); and Y is the crop yield from the same area (Hoekstra et al., 2011). Therefore, the WF_crop_ is a measure of WUE, and thereby agricultural practices that either lower the CWU or increase yields will decrease the WF (Hoekstra et al., 2011).

The Gray WF [GWF] was calculated following the methodology outlined by Hoekstra et al. (2011) and Franke et al. (2013). The GWF from agricultural fields, a non-point pollution source was calculated following equation:

(4) $$\begin{array}{l} \text{GWF}=\frac{\text{L}}{{\text{c}}_{\text{max}}-{\text{c}}_{\text{nat}}}\;\left[\text{volume tim}{\text{e}}^{-1}\right] \end{array}$$

where L is the pollutant load (kg), originating from an agricultural field of a determined size (ha) over a defined period of time (year) reaching a underground or surface water source; c_max_ is the maximum allowable concentration of the pollutant in the water source based on environmental regulations and standards; and c_nat_ is the natural or ambient concentration of the pollutant in the water source prior to pollution caused by human activity (Franke et al., 2013).

The WF_gray_, in distinction of GWF, is expressed per unit of product and is calculated similarly to WF_blue/green_ (Equations 1, 2), with GWF being used instead of CWU.

(5) $$\begin{array}{l} \text{W}{\text{F}}_{\text{gray}}=\frac{\text{GWF}}{\text{Y}}\;\left[\text{volume mas}{\text{s}}^{-1}\right] \end{array}$$

where GWF is the pollutant load divided by the difference between c_max_ and c_nat_ (see Equation 5); and Y is the crop yield (ton) (Franke et al., 2013).

In addition to dividing CWU or GWF (Equations 2, 3, 5) by the yield for the WF_crop_ calculation (ton ha^−1^) an additional parameter of water efficiency was used instead of Y: energy (10^6^ kcal ha^−1^. Data regarding nutritional values of crops (kcal) was based on the online USDA's National Nutrient Database [United States Department of Agriculture (USDA), 2015].

### WF_regional_ methodology

An additional WF measurement analyzed for this study is the regional, or area, WF, (*WFP*_*regional*_) that was calculated as of Equation (6):

(6) $$\begin{array}{l} \text{W}{\text{F}}_{\text{regional}}=\underset{\text{q}}{\sum }\text{W}{\text{F}}_{\text{proc}}\left[\text{q}\right]\;\left[\text{volume tim}{\text{e}}^{-1}\right] \end{array}$$

where WF_regional_ is calculated as the sum of the WF of the different studied processes (WF_proc_[q]) in the area of study (Hoekstra et al., 2011). In the case of this study, the process (WF_proc_) examined is crop cultivation. The WF_regional_ was calculated separately for each crop and for each drainage basin (Hoekstra et al., 2011).

### Data sources for WF_blue_ and WF_green_ calculation

As suggested by Hoekstra et al. (2011), the ET of the different crops were calculated using the CROPWAT model. This was done by multiplying ET_0_, i.e., the conventional reference evapotranspiration, based on a physical equation known as FAO56 Penman-Montieth equation, by different crop coefficients (k_c_) that were developed for most commercial crops (Allen et al., 1998). Climate data for the CROPWAT model was gathered from the Zemah Meteorological Station (ZMS), of the Israel Meteorological Service, located south of the Sea of Galilee, at 35°35′E longitude, 32°43′N latitude at an elevation of –200 m. Radiation and ET_0_, which are usually calculated by CROPWAT based on climate data were already calculated in the ZMS based on measured climatic parameters. Since sunshine hours was not measured in ZMS, it was inputted manually to reflect the radiation and ET_0_-values provided by ZMS. Average monthly climatic data used for the ET calculation are presented in Table 1. Climatic values for net-house banana were adjusted following results of local studies that measured the effects of net-houses on wind-speed and radiation (Israeli et al., 2012). Soil data, i.e., total available soil moisture (mm/meter), maximum rooting depth (cm), and maximum rain infiltration rate (mm/day) was based on Ravikovitch (1981) soil sampling data from the study area and the SPAW tool (Saxton, 2007). Additional WF calculations were performed based on soil samples from five different locations across the studied area, provided by the local agricultural R&D center (*Zemah Nisyonot*—ZN).

**Table 1:** **Climatic data used for the calculation of WF**.

**Month**	**Min. Temp (°C)**	**Max. Temp (°C)**	**Humidity (%)**	**Wind (km day^−1^)**	Sun^*^ (h day^−1^)	**Radiation (MJ m^−2^ day^−1^)**	**ETo (mm day^−1^)**
Jan.	8.6	17.8	74	125	4.8	9.6	1.5
Feb.	9.2	19.3	73	131	5.4	12.2	2.0
Mar.	10.4	23.0	69	132	7.5	17.3	3.0
Apr.	13.8	27.4	63	145	8.5	21.1	4.2
May	17.3	32.1	60	159	10.6	25.5	5.6
Jun.	20.8	35.3	59	174	11.9	27.8	6.7
Jul.	24.0	37.5	59	181	11.6	27.0	7.0
Aug.	24.5	37.5	61	174	10.7	24.6	6.5
Sep.	21.9	35.1	61	150	9.7	21.0	5.3
Oct.	18.5	30.9	60	125	7.9	15.7	3.7
Nov.	13.2	24.0	61	118	6.6	11.8	2.4
Dec.	10.1	19.8	70	124	4.9	9.1	1.7
Average	16.0	28.3	64	145	8.3	18.6	4.13

Monthly averages for the years 2000–2013. Sun hours were estimated based on measured radiation values (

* *estimated value)*.

**Figure 2:**
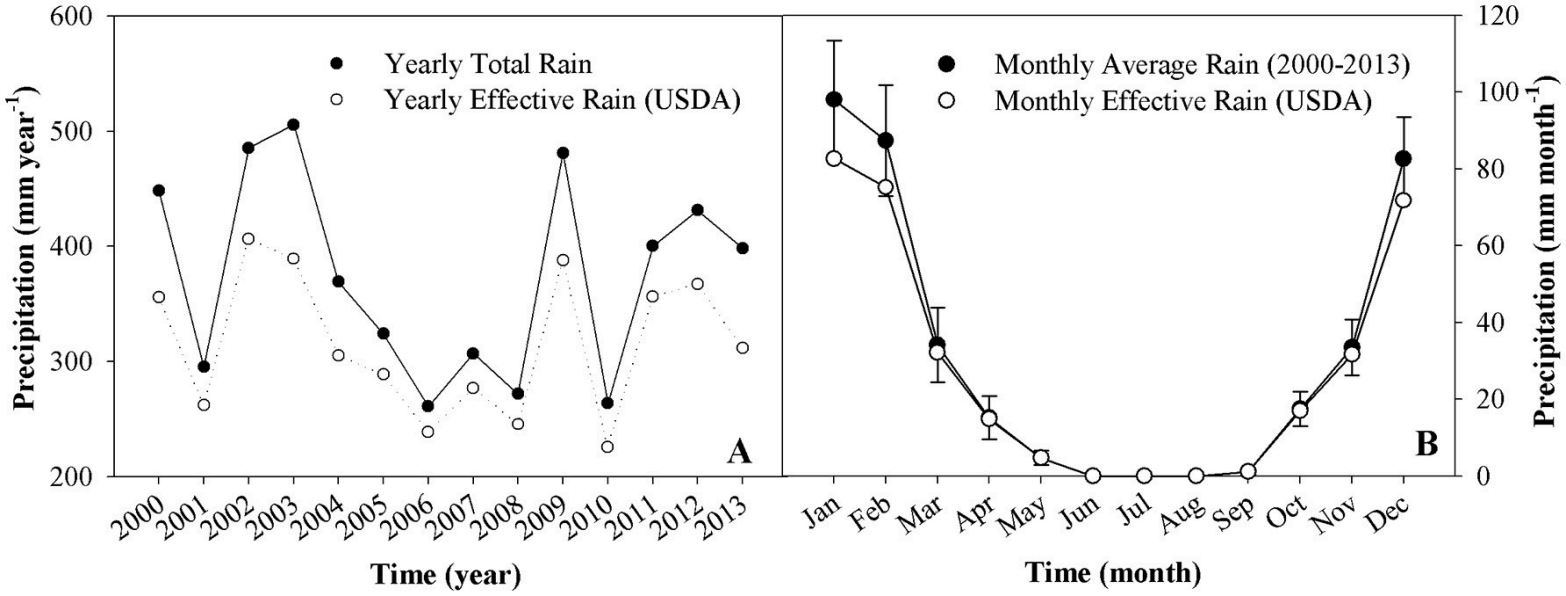
**Temporal changes in yearly and monthly precipitation and effective rain used for the calculation of WF_green_ and WF_blue_. (A)** Yearly total and effective rain for the years 2000–2013; **(B)** Monthly rain and affective rain averages for the years 2000–2013. Monthly rain averages are total ± *SE* (*n* = 14 years). Precipitation as measured in ZMS, and effective rain was calculated following WFAM (Hoekstra et al., 2011) using the USDA soil conservation service formula.

Rain data was collected for the years 2000–2013 from ZMS as daily values and was summed to provide monthly values. The monthly values for the years were averaged to provide the multiannual averages (IMS, 2016). While average rain levels were measured at 374 mm year, yearly variations range between 505 mm year^−1^ (135% of the average) and 260.9 mm year^−1^ (70% of the average) (Figure 2A). High variation in precipitation levels can be seen throughout each year, with the majority of precipitation concentrated in 3 months—December to February (Figure 2B). Effective rain, which represents the volume of rain-water that is not lost via deep percolation or runoff and is available for plant use, was calculated by CROPWAT using the USDA Soil Conservation Service method as suggested by Hoekstra et al. (2011).

### WF_gray_ methodology and data sources

The pollutants chosen for this study were N and P. The pollutant load reaching the water resources was calculated using suggested average leaching-runoff fractions (0.1 for N and 0.003 for P), which is the estimated percentage of the chemical substance that is lost to groundwater through leaching and to surface water through runoff (Franke et al., 2013). For each crop, GWF was calculated for both N and P, with the higher value of the two used for the WF_gray_ calculation. Ambient (c_nat_) and maximum allowable (c_max_) concentrations for the Sea of Galilee and Lower Jordan River are presented in Table 2.

**Table 2:** **Ambient and maximum allowable N and P concentrations in the Lower Jordan River and the Sea of Galilee [based on Berman, 1998; Hambright et al., 2000; Ministry of Health (MOH), 2013]**.

**Water source**	**Natural concentration (c_nat_)**		**Maximum concentration (c_max_)**	
	**N (mg L^−1^)**	**P (mg L^−1^)**	**N (mg L^−1^)**	**P (mg L^−1^)**
Lower Jordan River	0.7	0.02	10	1
The Sea of Galilee	0.7	0.02	1	0.038

The c_nat_ and c_max_ concentrations shown in Table 2 are based on measured concentrations of the Sea of Galilee for over six decades (Berman, 1998; Hambright et al., 2000). These concentrations are used as ecological indices for the management of the Sea of Galilee by the Israel Oceanographic and Limnological Research Institute who is responsible for monitoring the lake's chemistry and ecological health. As values were presented as ranges, the highest value was selected for a more conservative GWF estimation. Natural concentrations (c_nat_) for the Lower Jordan River were considered to be identical to its source water—the Sea of Galilee. Maximum allowed concentrations (c_max_) for the Jordan River were based on two data-sources that use the same standard: The first is the legally binding c_max_ for discharge of treated effluents into rivers, and the second is the suggested c_max_ for the Kishon River—the only recommended concentrations required for stream ecological rehabilitation published by the Ministry of Environment [MoE and KRA, 2002; Ministry of Health (MOH), 2010].

**Figure 3:**
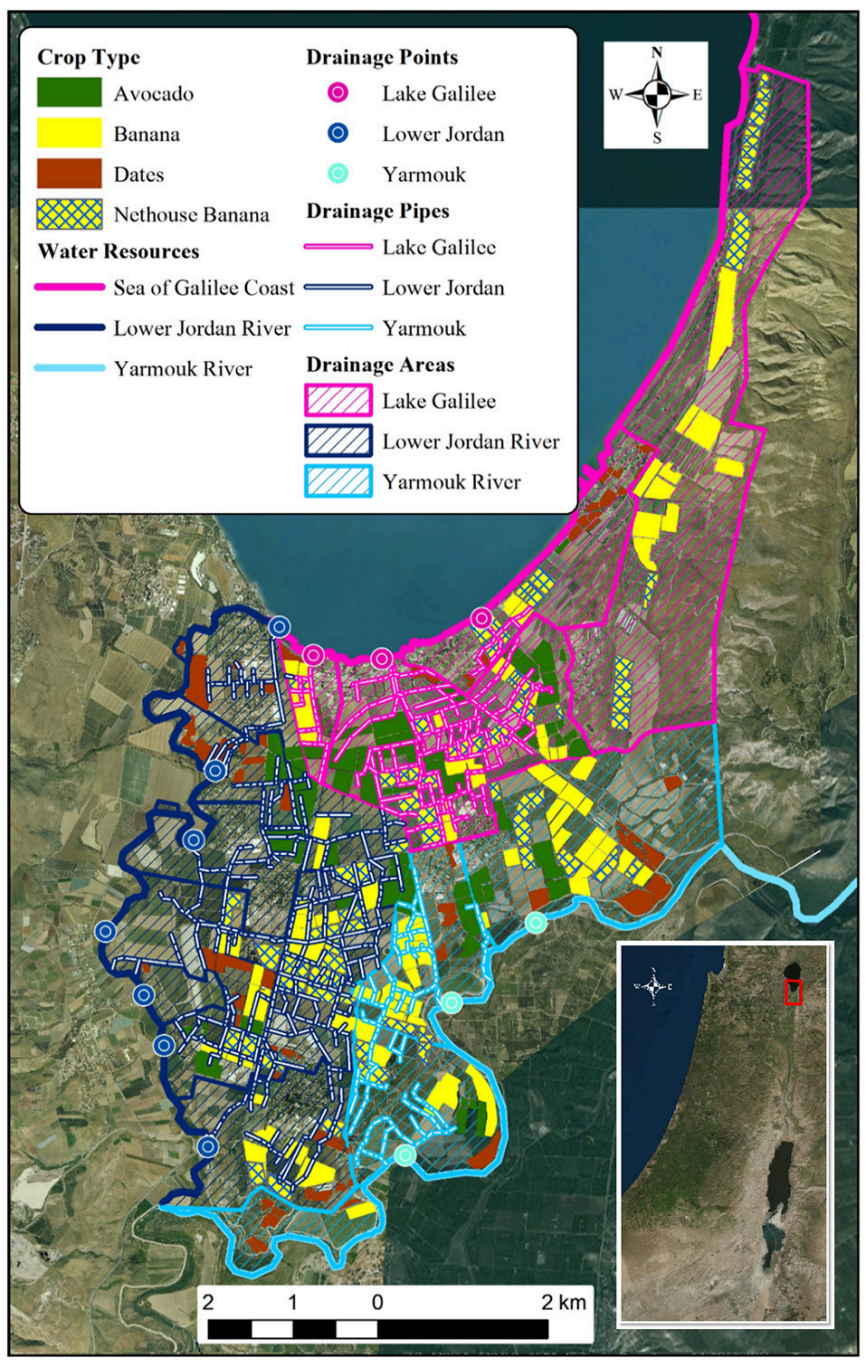
**Drainage zones in the area of study (superimposed of the distribution map of the main plantation crops in the area**. The drainage zones are determined by the drainage pipes system installed in the area and their discharge points (source: MOAG, ZN).

In order to determine the water body into which each field drains to, two drainage basins were defined—the Sea of Galilee Basin (SGB) or the Lower Jordan River Basin (LJRB). A GIS map of the extensive drainage system installed during the 60s beneath the studied area was used (Figure 3). The drainage area was determined according to the drainage point of each group of pipes, and where no drainage pipes were available geographic proximity of water body was used instead. The Yarmouk River was treated as part of the LJRB. Once the area of each basin was delimited, each agricultural field was assigned with a drainage basin that determined which concentrations to be used for its GWF calculation.

Additional data regarding average irrigation was based on irrigation recommendation tables developed by the Agricultural Extension Service (AES) of the Ministry of Agriculture and Rural development of Israel (MOAG) (Agricultural Extension Services (AES) and Eizenkott, 2014). These tables are comprised of three coefficients per month, constructed for successive 10 days groups, which are multiplied by the measured daily ET_0_ from the nearest meteorological station for irrigation depth to be applied. These coefficients are the results of local research and experiment and include, in banana crops for example, extra water for leaching salts from the soil. This data was used to compare to the WF results as calculated by the methods outlined above. Additionally, fertilization and yield for each crop were based on AES data sheets and interviews with local AES agricultural advisors and of the local agricultural R&D center (*Zemah Nisyonot* -ZN) which work closely with farmers and provide recommendations for irrigation and fertilization practices. Fertilization levels used for the GWF calculations are presented in Table 3.

**Table 3:** **Fertilization application levels in the area of study based on local recommendations (by the Extension Service of the Ministry of Agriculture) and estimated amounts leached into water resources per ha based on Franke et al. (2013) leaching coefficients**.

**Crop**	**Element**	**Application (kg year^−1^)**	**Total N/P (kg year^−1^)**	**Estimated total leached (kg year^−1^)**
Banana	N	300	30.0	3.0
	P (as P_2_O_5_)	42	18	0.55
Dates	N	25	25	2.5
	P (as P_2_O_5_)	0	0	0
Avocado	N	371	371	37.1
	P (as P_2_O_5_)	50	22	0.66

## Results

The study was conducted in two main steps. First, the WF_crop_ (blue, green, and gray) of the four main plantation crops in the area of study (Open-field and net-house banana, palm dates, and avocado) were compared. Comparisons between estimated crop ET and recommended irrigation values and between calculated WF (m^3^ ton^−1^) in the Jordan Valley area and global estimated values, are presented as well. Next, the WF_regional_ for each crop and for each drainage basin was calculated for the entire study area. Additionally, a scenario showing the potential of modeling changes to test their impact on the regional WF, was evaluated by testing the impact of replacing all open-field banana with net-house banana.

### Crop WF

#### Selection of temporal scale for WF_blue+green_ calculations

**Figure 4:**
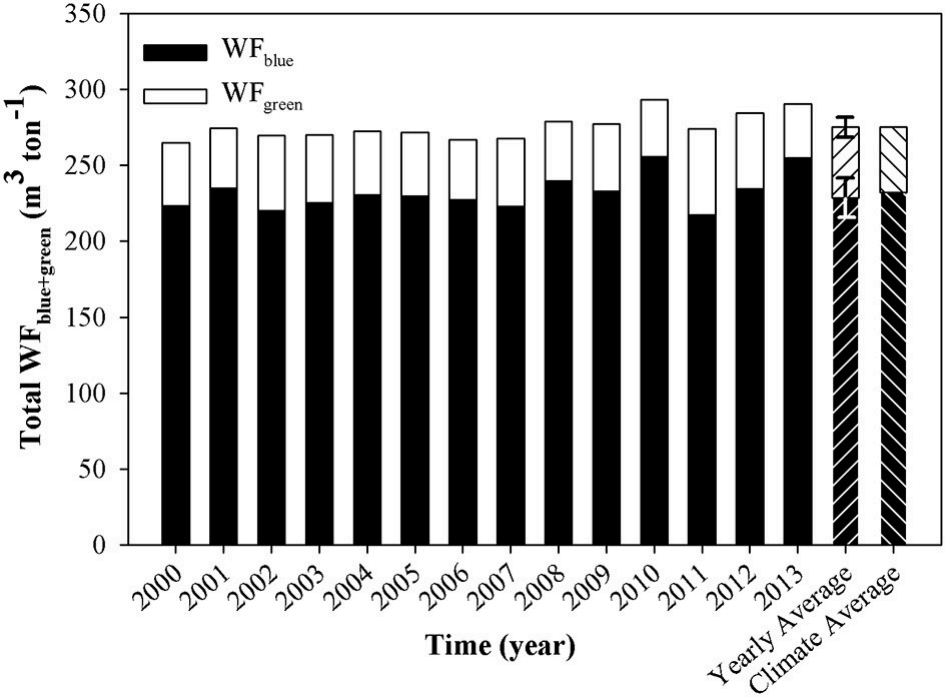
**WF_blue+green_ for open-field cultivated banana for the years 2000–2013**. Average of the results for the 14 analyzed years ±*SD* (*n* = 14) based on climatic data for each year (“Yearly Average”), and based on average climatic data of the entire period (“Climate Average”) are presented in the bars on the right hand side.

WF_blue/green_ for open-field cultivated banana was similar throughout the 14 analyzed years (2000–2014), displaying an average (“*Yearly Average*”) with a small standard deviation (Figure 4). Accordingly, WF_blue/green_ results calculated based on the average of the climate parameters for the entire study period (Table 1—“*Climate Average*”) were very similar (differed by <4 m^3^ ton^−1^) to the WF_blue/green_ results calculated based on the *Yearly Average*. In order to provide WF results that reflect long term multiannual patterns, all subsequent results presented used the *Climatic Average* for the calculation of ET and WF.

#### ET and biomass and energetic blue/green WF for the studied plantation crops

**Figure 5:**
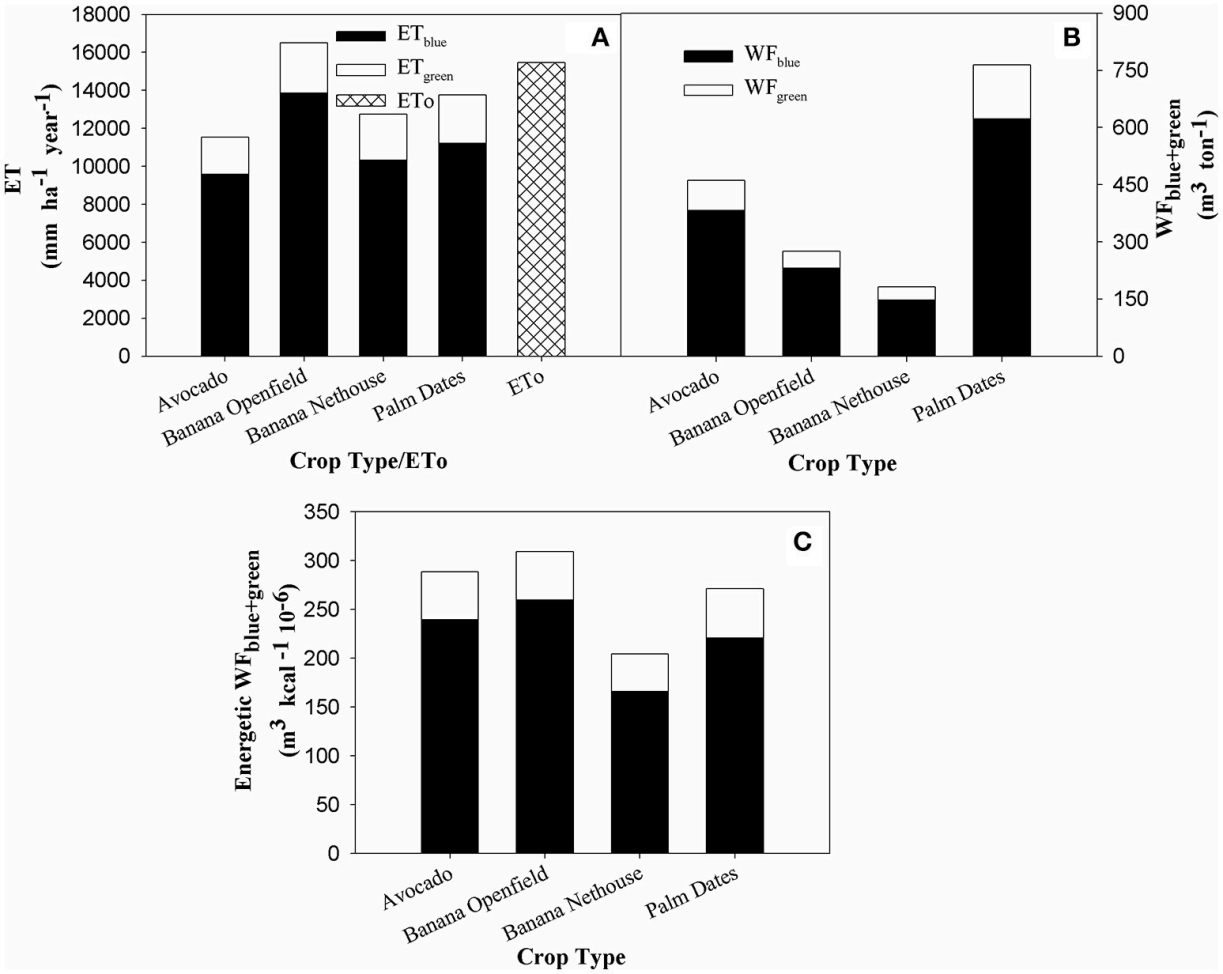
**Evapotranspiration and Blue/Green WF for main plantation crops in the Jordan Valley: banana cultivated in net-houses or in open-fields, palm-dates, and avocado**. Results are based on average of ZMS climatic data for the years (2000–2013). **(A)** Yearly Blue and Green ET and ET_0_ measured in ZMS per ha; **(B)** Blue and Green WF (m^3^ ton^−1^); **(C)** Energetic Blue and Green WF (m^3^ kcal^−1^ 10^−6^).

ET/WF_blue+green_ results of a CROPWAT analysis for the four major crop categories in the Jordan Valley show that the blue component has a much more significant role in the results compared to the green component (Figures 5A–C). ET_blue+green_ (m^3^ ha^−1^ year^−1^) for the studied crops range from 11,540 (avocado) to 16,507 (banana open-field, the only crop with higher ET compared to ZMS measured ET_0_) (Figure 5A), and WF (m^3^ ton^−1^) values range from 182 (banana net-house) to 765 (palm dates). The crop with lowest results for all three WF parameters is banana net-house (Figures 5A–C). On the other hand, while palm dates WF_blue+green_ (m3 ton^−1^) is 2.75 times higher than banana open-field (Figure 5B) it is has second lowest energetic WF (Figure 5C).

#### Gray WF for Jordan Valley and sea of Galilee basins

**Figure 6:**
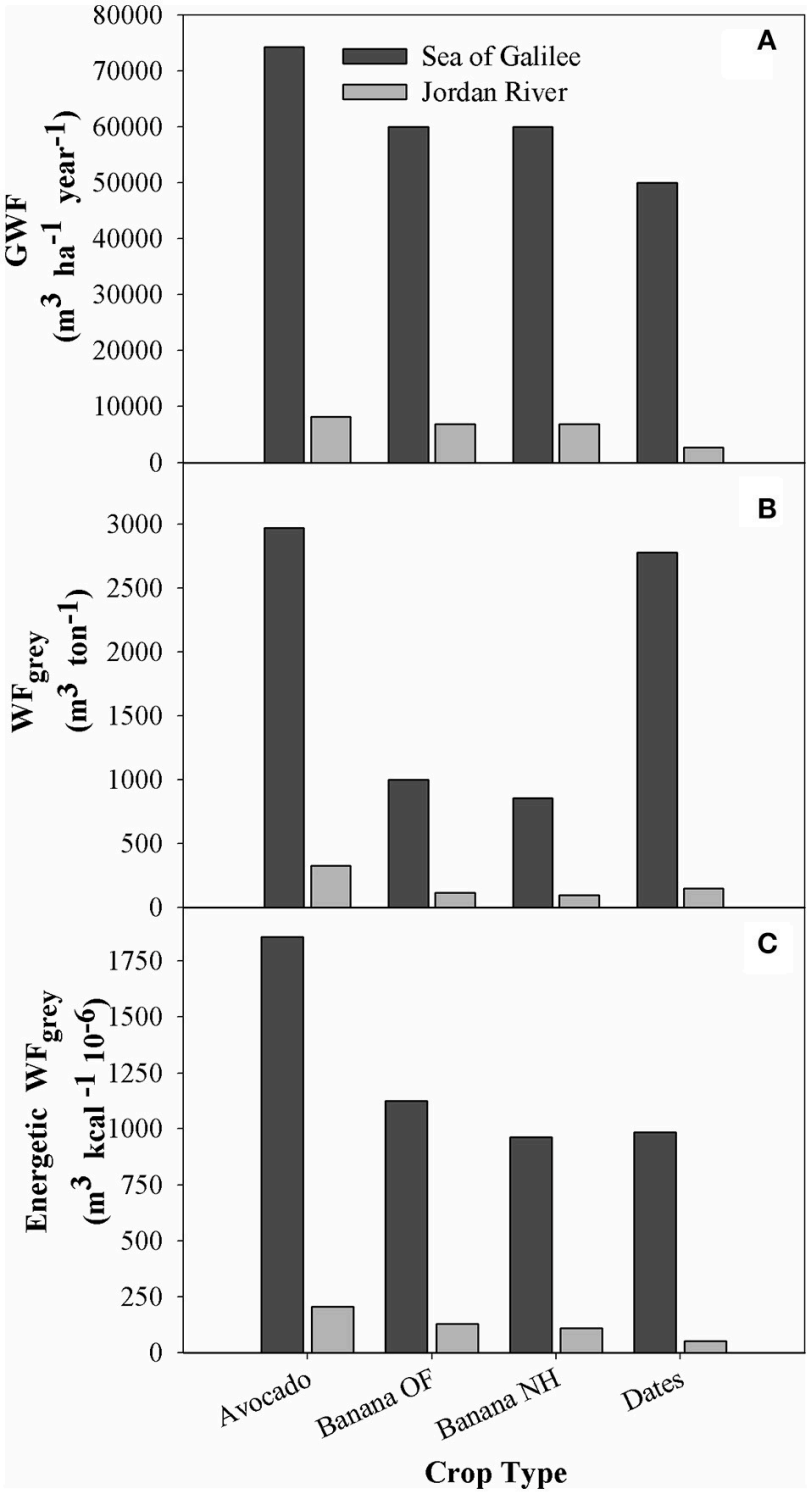
**Gray Water volume and Gray WF for major plantation crops in the Jordan Valley**. Comparison of crops draining to Sea of Galilee and to the Jordan River. **(A)** Yearly Gray Water volume per ha; **(B)** Gray WF (m^3^ ton^−1^); **(C)** Energetic Gray WF (m^3^ kcal^−1^ 10^−6^).

The WF_gray_ results were divided according to the two separate drainage basins in the area of study. The difference between the allowable maximum concentrations is the only parameter that changes between the two regions, with ambient concentration and pollutant load remaining identical for each crop. For all crops analyzed, values for the Sea of Galilee drainage basin are distinctly higher than those of the Jordan River (Figures 6A–C), this is due to the stricter maximum concentrations required for the lake (10 times lower for N and 26 times lower for P, see Table 2).

GWF (m^3^ ha^−1^ year^−1^) values range from 74,250 (avocado) to 50,000 (palm dates) for the SGB and 8188–2688 for the LJRB (Figure 6A). The WF_gray_ (m^3^ ton^−1^) results, on the other hand, show that the banana crops have a significantly lower WF compared to the two other crops (Figure 6B). Results for all different parameters emphasize the large gap between the high SGB WF compared to the relatively low results in the LJRB (Figures 6A–C). Similar to the results seen in Figure 5, palm dates have a high WF_gray_ (m^3^ ton^−1^) (Figure 5B) but a low energetic WF_gray_. For LJRB, the results for palm dates are even lower than for net-house banana, that otherwise remains with the lowest WF_gray_ (Figures 5B,C). The results shown in Figures 5B,C further emphasize the influence of the parameter chosen for the WF calculation on the results. The WF_gray_ of palm-dates, a low yield crop, is highest when dividing water usage by yield weight, but when water usage is divided by energy produced its WF drops due to the crop's relative high energy content.

#### Comparison to local irrigation recommendations and global results

**Figure 7:**
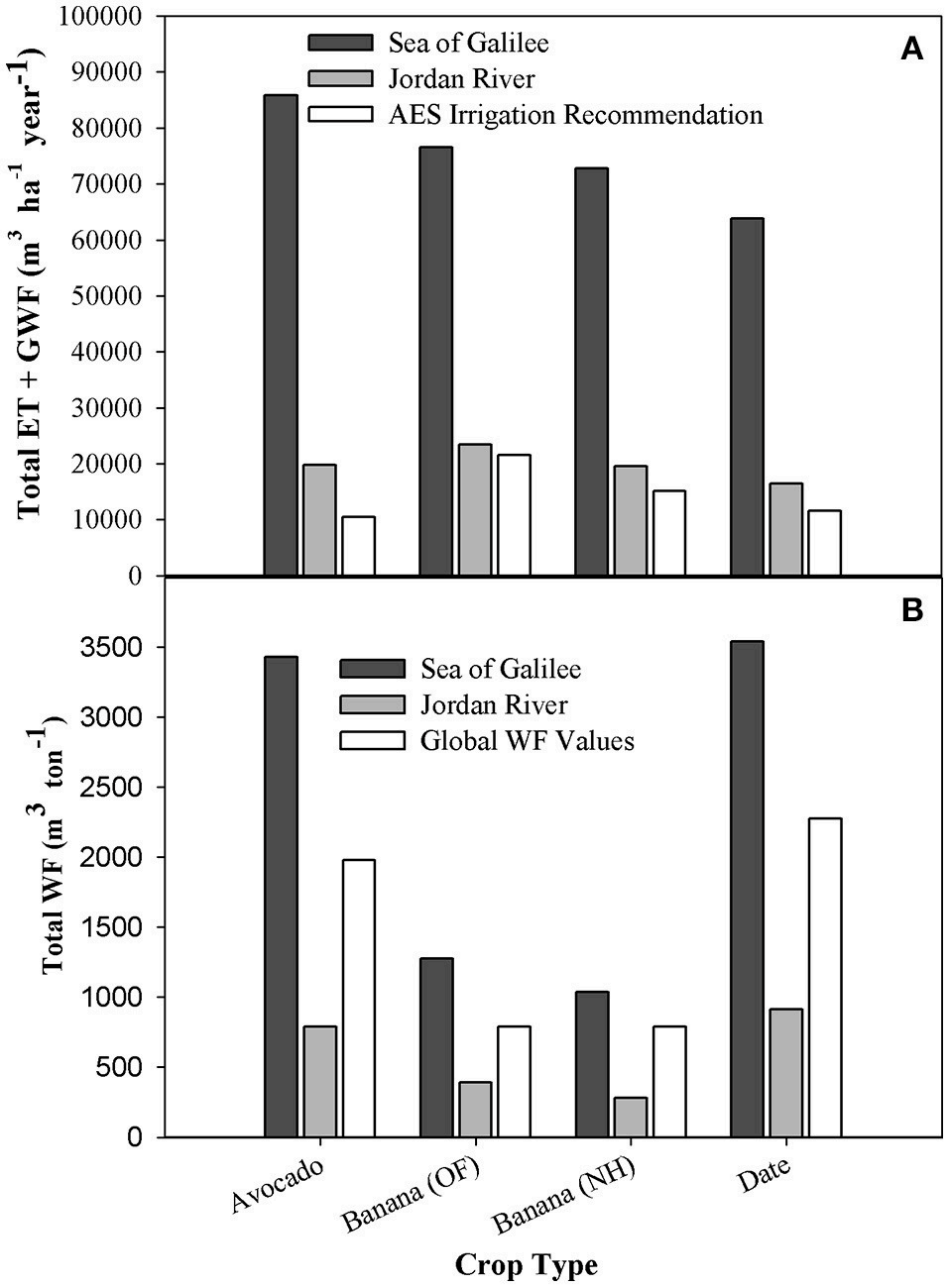
**Total ET+GWF, AES Irrigation recommendations, total WF, and global WF-values major plantation crops in the Jordan Valley**. Comparison of plantations draining to Sea of Galilee and to the Jordan River. **(A)** Yearly Total ET and Gray Water compared to AES regional irrigation recommendation per ha [Agricultural Extension Services (AES) and Eizenkott, 2014]; **(B)** Total WF and global WF-values (m^3^ ton^−1^) (Mekonnen and Hoekstra, 2011).

Comparing ET+GWF (which combine the results of Figures 5A, 6A) to the AES irrigation recommendations illustrate a huge difference from the SGB results—ranging from 815% (avocado) to 356% (banana open-field) of AES equivalent. In comparison, the LJRB results are higher than the AES recommendations by 88% for avocado and only 8% for banana open-field (Figure 7A). It is important to note that AES recommendation values do not include rain water (green) and although they do include additional water for leaching salts from the soils, these are much lower than the GWF presented in Figure 6A especially compared to SGB GWF.

Table 4 details the different components of the WF (m^3^ ton^−1^) results, allowing a better understanding of each component's influence on the results. When looking at the WF_blue+green_ for main plantation crops in the Jordan Valley alone, the results are significantly lower than the global values (between 61 and 35%). When the WF_gray_ of the LJRB crops are added to WF_blue+green_, values remain between 1.4 and 2.5 times lower than their global equivalents. Adding the WF_gray_ of the SGB crops, on the other hand, results in a WF_total_ that is between 1.4 and 1.9 times higher than the global WF.

**Table 4:** **Calculated Blue, Green, and Gray WF-values for the Jordan Valley, compared to global published values (Mekonnen and Hoekstra, 2012)**.

**WF component (m^3^ ton^−1^)**		**Banana**			**Avocado**		**Palm-dates**	
		**Net-house Jordan valley**	**Open-field Jordan valley**	**Open-field Global**	**Jordan valley**	**Global**	**Jordan valley**	**Global**
Blue		231	383	97	383	283	622	1250
Green		44	79	660	79	849	142	930
Subtotal (Blue+Green)		275	462	757	462	1132	764	2180
Gray	Jordan River	98	115	–	328	–	149	–
	Sea of Galilee	857	1000	–	2970	–	2778	–
	Global	–	–	33	–	849	–	98
Total (Blue + Green + Gray)	Jordan River	373	577	–	790	–	913	–
	Sea of Galilee	1132	1462	–	3432	–	3542	–
	Global	–	–	790	–	1981	–	2278

*WF_gray_ results displayed for both drainage basins*.

### Regional WF

**Figure 8:**
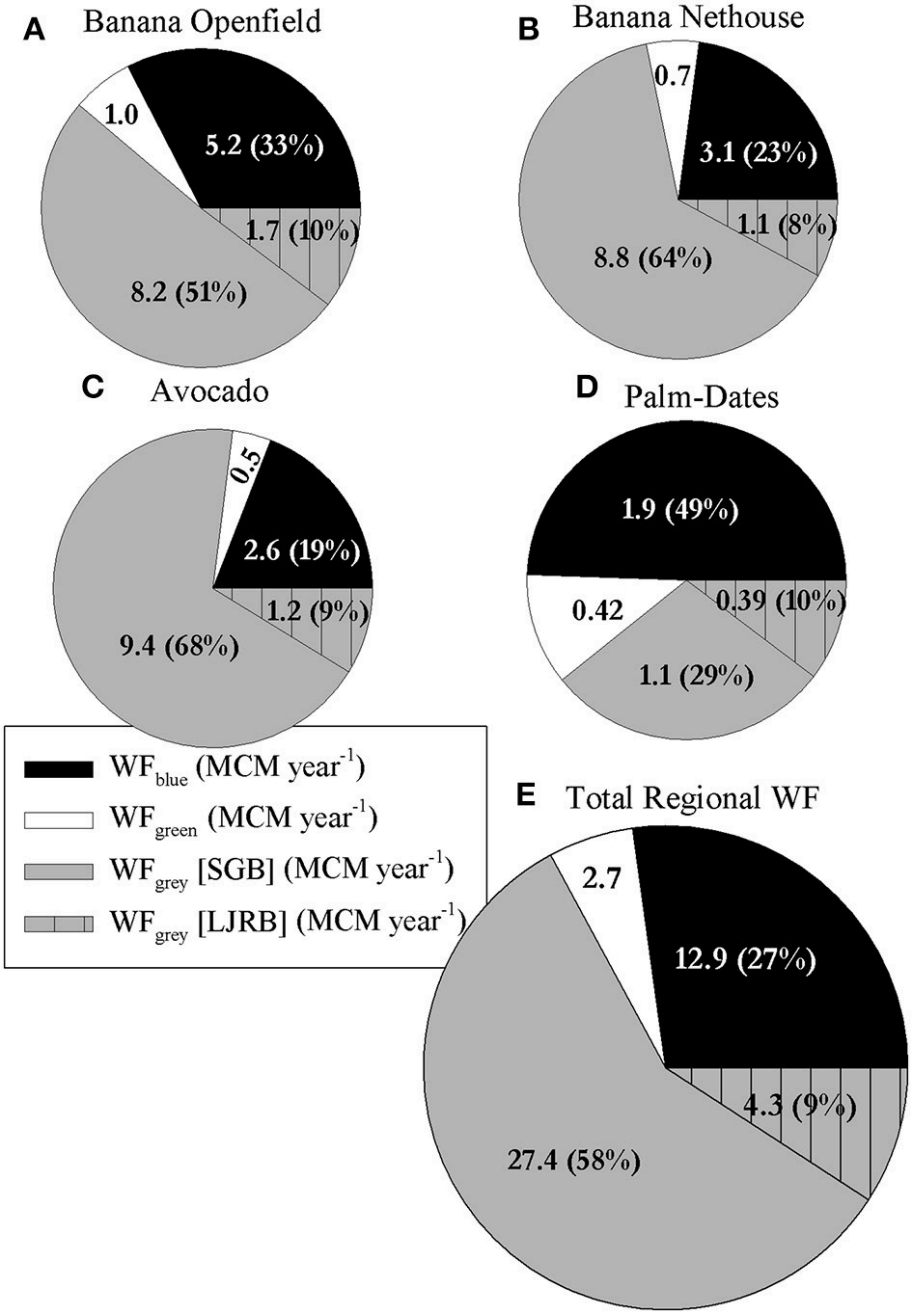
**Distribution of the regional annual WF for each individual studied crop, and the total regional WF, into Blue, Green, and Gray WF. (A)** Open-field banana (378 ha in the area of study); **(B)** Net-house banana (304 ha); **(C)** Avocado (274 ha); **(D)** Palm-Dates (166 ha); **(E)** Total Regional WF of the studied area (1122 ha). Values in the charts are MCM year^−1^. The gray WF of each crop is divided to the drainage to the Sea of Galilee and the Jordan River.

In addition to calculating the WF_crop_, the WF_regional_ was calculated as well. These results indicate the total yearly WF or water-use (see Equation 6) for each crop in the entire study area (Figures 8A–D) and the total for all crops (Figure 8E). Palm-dates, with only 13% of the crop grown in the in SGB, is the only crop whose WF_regional.gray_ is lower than the WF_regional.blue+gray_ (Figure 8D). For the three other crops, the WF_regional.gray_ is the largest component—between 51 and 68% of the total WF_regional_ (Figures 8A–C). For the entire studied area, 67% of the WF_regional_ is comprised of the WF_regional.gray_ component—out of which, almost 87% (58% of total) is derived from crops grown in the SGB (Figure 8E).

**Figure 9:**
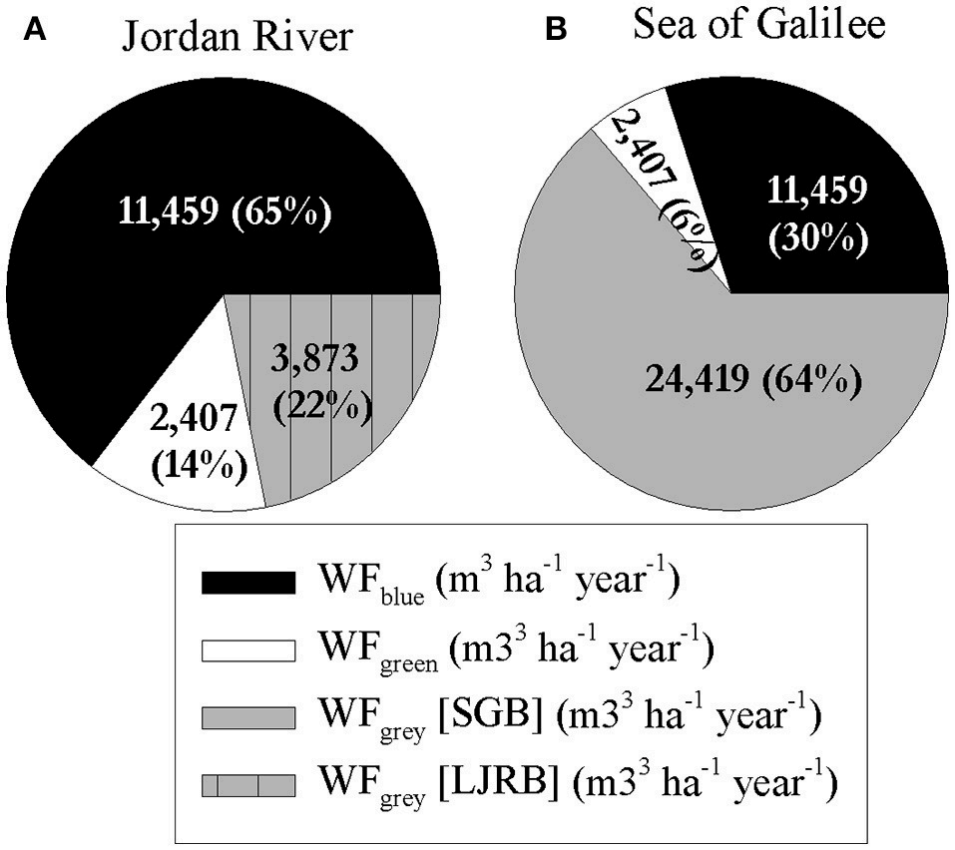
**Average WF per hectare for main plantation crops in Jordan River and Sea of Galilee drainage basins (m^3^ ha^−1^ year^−1^) [total ha]. (A)** Jordan River average WF per ha (430 ha); **(B)** Sea of Galilee average WF per ha (692 ha).

Dividing the WF_regional_ of the SGB and LJRB by the total area of each basin gives the average WF (m^3^ ha^−1^ year^−1^) in each basin. In the LJRB, the average WF_gray_ is a third of the WF_blue_ which comprises 65% of total WF (Figure 9A). In the SGB, however, the average WF_gray_ is more than double the WF_blue_ and 64% of total WF. The average WF_gray_ in the SGB is 20,546 m^3^ ha^−1^ year^−1^ higher than the average WF_gray_ in the LJRB.

### Example of modeling changes to WF using GIS

In addition to analyzing the current situation, using GIS enables to easily compute the effect of potential changes in cultivation practices on the WF. Two examples of such computations made as part of this study are presented: (1) A change of cultivation practice; and (2) A change in fertilization levels.

**Figure 10:**
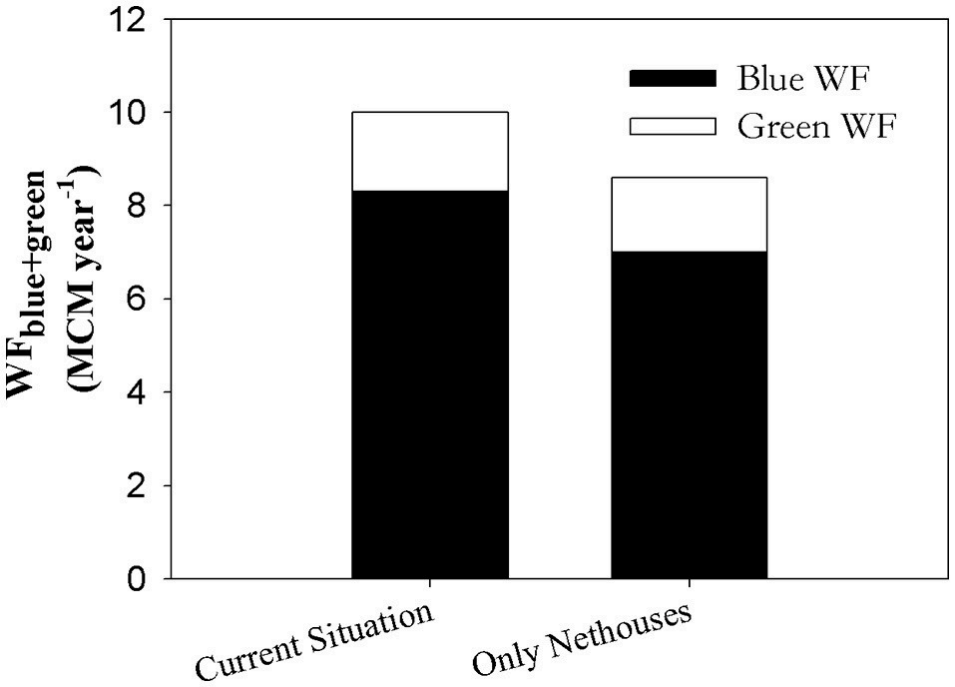
**WF_regional.blue+green_ (MCM year^−1^) of banana (net-house+open-field) in the current situation and modeled situation where all open-field banana fields were replaced with net-house banana**.

The first scenario examined the effect of replacing the 377.8 ha of open-field banana with net-house banana, bringing the total banana net-house area to 681.6 ha. This was done by redefining crop type from “open-field” to “net-house” for all “open-field banana” polygons and recalculating WF_regional_ based on the new configuration. This resulted in a reduction of 1.3 MCM year^−1^ WF_regional.blue_ and 0.1 MCM year^−1^ in the WF_regional.green_ (Figure 10).

In the second scenario, GWF calculations were done based on 10% and 20% higher and lower fertilization levels (Table 5). Reducing fertilization levels by 20% resulted in a total of WF_regional.gray_ of 25.4 MCM year^−1^ or 6.3 MCM year^−1^ (out of which 5.5 MCM year^−1^ were in the SGB) lower than the current situation.

**Table 5:** **The effect of changes in fertilization rates on the volume of Gray Water produced (m^3^ dunam^−1^ year^−1^) current fertilization levels are based on data obtained from AES**.

**Crop type**	**Drainage area**	**20% less fertilizer**	**10% less fertilizer**	**Current amount**	**10% more fertilizer**	**20% more fertilizer**
Banana	Sea of Galilee	4800.0	5400.0	6000.0	6600.0	7200.0
Banana	Jordan River	550.2	619.0	687.8	756.6	825.3
Avocado	Sea of Galilee	5940.0	6682.5	7425.0	8167.5	8910.0
Avocado	Jordan River	655.0	736.9	818.8	900.7	982.5
Palm-dates	Sea of Galilee	4000.0	4500.0	5000.0	5500.0	6000.0
Palm-dates	Jordan River	215.1	241.9	268.8	295.7	322.6

## Discussion

The present study evaluated potential benefits of combining the WF methodology with high spatial resolution local data using the Jordan Valley region as a model system of study. This was facilitated by the availability of a GIS vector-layer that included high resolution spatial data for crop distribution, drainage basins and agricultural practices, and additional spatial data collected from local agricultural advisors which included fertilization and irrigation practices. The high resolution of the data, combined with the use of GIS, allowed assessing the impact of agronomic parameters on the WF. Additionally, the existence of a GIS layer of an underground drainage system installed beneath the study area allowed a high-resolution analysis of local difference to the GWF. These differences are significant when nearby agricultural fields drain to separate water bodies with significant difference in the maximum allowed concentration. In this case study, field draining into the highly-sensitive Sea of Galilee have a much higher GWF compared to proximate fields that drain into the Jordan River.

The following sections discuss the benefits of integrating the WF methodology with high spatial resolution local factors with the use of GIS and analyze the crop WF results for the studied area, focusing on the influence of factors such as cultivation methods (open-field vs. net-house cultivation) extent of N and P fertilization and drainage basins.

### Crop WF

The study focused on the crops banana (open-field and net-house), avocado and palm-dates, which together represent 52% of all cultivated land in the area of study (Figure 1). While net-house banana displayed the lowest WF in all categories, asserting the benefits of net-house in terms of increasing WUE, a closer look at the results provide a more complex picture. Palm-dates has the highest WF (m^3^ ton^−1^) of all analyzed crops (Figure 5B), however for the energetic WF it is second only to banana net-house (Figure 5C). On the other hand, open-field banana, with the second lowest WF (m^3^ ton^−1^) has the highest energetic WF (m^3^ kcal^−1^ 10^−6^) (Figures 5B,C). The trend for WF_gray_ is similar, but for the LJRB, palm-dates results are lower than even net-house banana for the energetic WF (Figures 6B,C).

These results emphasize the drawback of using the efficiency of water use per yield production [m^3^ water (ton yield)^−1^] as an exclusive WF unit. Although calculating the WF based on the yield of the crop produced for each m^3^ of water consumed is sensible when comparing crops with similar yields, it is more problematic when comparing high-yield crops vs. low-yield crops. While, banana cultivated in open-field produces a higher yield per m^3^ than palm-dates, it also produces less energy compared to palm-dates. Water efficiency is treated differently depending on the discipline and the purpose of the study. Using additional parameters of water efficiency when comparing different crops provides a more nuanced view on the WF of a specific crop (Goncalves et al., 2007; Pereira et al., 2012; Nair et al., 2013). Nutritional value (calories or others) (Figures 5C, 6C), which is tied to the yield, is an additional factor that should be taken into consideration when determining the suitability and benefits of specific crops and cultivation-practices on water-saving. Additional factors that should be taken into consideration in future studies of this type include the financial WF, the profit earned for each m^3^ used for the different crops, comparison of food crops and non-food-crops with an emphasis on food security, type of water used (treated waste water, saline water, freshwater, etc.) risk factors, weather factors, and additional local cultural dimensions. These aspects, that influence the crop choice, must be all incorporated when developing an agricultural policy that based on technical, financial, ecological and social factors.

#### Effects of cultivation practices and drainage basin on WF

Interestingly, the crop with the lowest WF_blue+green_ for both parameters analyzed, was net-house cultivated banana (Figures 5B,C). The WF _blue+green_ of banana net-house is 34% lower than the yield (Figure 5B) and energetic (Figure 5C) compared to banana open-field. The utilization of net-house can therefore be translated into a saving of ~93 m^3^ ton^−1^ for growing bananas (Figure 5B). The net-house benefits are caused by lowering the actual water requirement of the crop caused by reduction of radiation and wind-speed (Tanny et al., 2003; Tanny, 2013). Additionally, certain net-houses provide protection from insects and climatic damages. The combined effect of cultivating bananas in the Jordan Valley under net-houses is both a decrease in evapotranspiration, and an increase of the yield by about 10 ton ha^−1^, thus lowering the WF compared to open-field banana cultivation (Or et al., 2011). These benefits were further emphasized by the estimated reduction of 14% in the WF_regional.blue+green_ of banana when considering full utilization of net-houses in the study area (Figure 10).

To the best of our knowledge there exists only one study dealing with the influence of cultivation practices on the WF. This study showed a reduction of WF of tomatoes in Spain when grown in greenhouses compared to the open-field (Chapagain and Orr, 2009). These results, join research that highlights the potential influence of the implementation of different agricultural practices such as net-houses, ground cover or irrigation water type on the WF of different crops and the need for additional comparative studies (Chukalla et al., 2015). Quantifying the reduction of the WF can help policy makers and farmers form well-rounded and informed decisions that integrate the costs and benefits of implementation of different agricultural practices. This may be done as part of wider policy effort to reduce crop WF by increasing the water and energy productivity of growing agricultural crops (Khan et al., 2009).

Although any pollutant can be used for GWF calculation, this study focused on the fertilizers N and P. Sea of Galilee, into which almost 40% of the fields in the study area drain, suffers occasional high levels of P, or more specifically, a low N:P ratio, that is considered to be related to outbreaks of cyanobacteria (Hambright et al., 2001; Hadas et al., 2015). Moreover, the Southern Jordan River is undergoing a rehabilitation effort, which will require reducing the high levels of N in the water (Gafny et al., 2010).

In our study, while the Sea of Galilee water quality standards used were based on extensive and specific research on the lake, the standards used for the Jordan River reflect a more generic approach to environmental standards of streams. While Israel law does not have general environmental requirements for freshwater quality (only for drinking), it does regulate the concentrations allowed for the release of effluents from sewage treatment plants to rivers [Ministry of Health (MOH), 2010]. These concentrations were used as the c_max_ for GWF calculations for this study. In practice, these concentrations are effectively used by the National Parks Authority as a baseline requirement for water quality for streams in Israel, and were therefore also suggested for the required concentrations for the Kishon River. The concentrations for Sea of Galilee, are based on more than 60 years of observations, and are considered by the Israel Oceanographic and Limnological Research, the national research institution that is responsible for managing the lake, to be environmental standards required for maintaining the ecological health of the lake (Berman, 1998; Hambright et al., 2000; MoE and KRA, 2002).

Crops draining into the Sea of Galilee were found to have a GWF that is almost 9 times higher for banana and avocado and 18 times higher for palm-dates compared to the same crops draining into the Jordan River (Figure 6A). This large difference was caused by the different maximum allowable pollution concentrations in the various basins, as the pollutant load (per ha in the agricultural field) and ambient concentration of the receiving water bodies used for the GWF calculation were identical in both drainage basins.

The large gap between the WF in the two drainage basins emphasizes the importance of determining natural and maximum acceptable concentrations for different water bodies for the purpose of calculating GWF results. European and global required environmental concentrations for freshwater resources vary between European countries and to a larger degree globally. The difference in standard global standards may suggest difficulties in global GWF comparisons, where countries with less stringent requirements may “benefit” from lower GWF results. Indeed, a challenge for GWF calculation and the environmental management of freshwater resources requires the development of scientifically-based standards, and to the very least a global base-line standard (Laane, 2005; Laane et al., 2005; Liu et al., 2012). Even so, while the GWF may be problematic when comparing between different countries, it can be useful in providing a baseline estimate for the “hotspot area” within a specific area or country. The GWF can serve as a useful, albeit not highly accurate, indicator for where further resources are required to be invested to quantify and mitigate the influence of pollutants on natural water resources.

Although, a few studies exist that compare the influence of drainage basins on the WF (Zeng et al., 2012; Schyns and Hoekstra, 2014; Vanham and Bidoglio, 2014), to the best of our knowledge, this study is the first to demonstrate the sensitivity of the GWF of agricultural systems to the drainage basin's water resource accepted concentrations.

#### Comparison to global results

Comparing the obtained WF to global values, highlights the impact of WF_gray_ on the results. The global WF_total_ of the studied crops was 2–2.8 times *higher* than the LJRB WF_total_. However, compared to the SGB crops the global values are 3.2—4.3 times *lower* than their global counterparts. Disregarding the WF_gray_ and comparing only the WF_blue+green_ results shows the studied area WF_blue+green_ are 2.5–4.2 times lower than their global counterparts (Figure 7B). Further, comparison of the specific WF components show that the local WF_blue_ component is higher than its global counterparts for all crops except palm dates, and the WF_green_ is significantly lower for all crops (Table 3). These results emphasize the dependence on intensive-irrigation in the studied region, which is necessary due to low precipitation levels (~360 mm year^−1^ on average, Figure 2A) and rainfall distribution over the year (long dry season with no precipitation and concentrated rainy season, Figure 2B). The comparison of the global and local results also highlights the high efficiency of water-use in the studied region, especially when WF_gray_ is not taken into account. More importantly, the huge discrepancies between the local and global average values emphasize the limited applicability of the global WF results and the importance of conducting local studies for a more accurate WF calculation (Lazzara and Rana, 2010).

### Regional WF

The final factor that was examined was the total WF_regional_, i.e., the total volume of the different water components used in the study area for each crop separately and combined. The WF_regional.total_ highlights, once again, the heavy influence of the WF_gray_ on the results. The WF_regional.blue_ constitutes of 83% of the WF_regional_._blue+green_, a value which is reduced to 27% (WF_regional.green_ equals 6% of WF_regional.total_) when adding the WF_regional.gray_ (Figure 8). This is caused mostly by the SGB WF_gray_ which constitutes of 58% of WF_regional.total_ (LJRB WF_regional.gray_ consists 9% of WF_regional.total_). The regional WF results suggest that the defining factor for crop GWF, and in certain cases also for the WF_total_, is foremost the crop's spatial location, or more specifically the water quality standards, i.e., the ecological sensitivity of the water body to which the pollutants in the cultivation area are drained. This can be best seen by comparing the average WF (m^3^ ha^−1^ year^−1^) for SGB and LJRB (Figure 9).

### The potential of GIS—WF integration

In the present study, the use of GIS facilitated an easy association between polygons representing actual agricultural fields with spatial parameters that influence the WF (e.g., drainage basin, soil type, climatic conditions, and precipitation levels). Thus, the influence of each parameter on the WF can be calculated and assigned to the relevant polygons. Furthermore, since GIS is a spatial platform, the total WF of the area can be easily calculated while taking into account the specific WF of each polygon based on the aforementioned parameters. This allows to analyze the degree of impact for individual parameter on the WF, such as exemplified in this research by the effect of the drainage basin on the WF_gray_ results.

### Increasing the spatial resolution of WF studies

The availability of the high-resolution data of the different parameters, including the drainage system installed under the studied area allows a high-spatial resolution with minimal use of estimations. There are only a few known studies combining WF and GIS. Three notable examples include a dissertation that calculated the WF of different river basins around the global (Hoekstra et al., 2011); a study in the Middle East that made use of global datasets and a much larger spatial resolution (0.5°) (Daccache et al., 2014); and a WF analysis of two river basins in Greece (Marini et al., 2015). Earlier studies used GIS to assess the spatial-temporal effects on crop productivity in the Philippines (Ines et al., 2002). The relatively low-resolution of the studies allows for important, albeit general, conclusions regarding regional water consumption but is less useful for assessing the impact of local climate, soil, growth practices which have, as shown in the results presented above, a significant impact on the WF. Studies on the water scarcity footprint of potato cultivation practices in Britain have already shown that local difference exist within a nation and even within a drainage basin (Hess et al., 2015). Therefore, in order to better translate research results into practical management practices, higher spatial resolution studies must be pursued.

Additional research is required in order to further increase the spatial resolution and promote the WF-GIS integration. A crucial step toward the integration of GIS with WF for high spatial resolution results is the development of local crop and soil coefficients since soil type strongly influences both ET and the rate of pollutant leaching (Gaines and Gaines, 1994; Djodjic et al., 2004; Katerji and Mastrorilli, 2009). Using crop coefficients based on local research and lysimeter and eddy covariance experiments can also help increase the accuracy of the crop ET estimation (Lazzara and Rana, 2010). Additional data from lysimeter experiments, such as leachate concentration, instead of rough estimates, can improve the accuracy of WF_gray_. Research regarding the impact of additional factors such as local agricultural practices (mulching or green manure), water type (e.g., freshwater, saline, and treated waste water) can further increase the accuracy and applicability of WF studies.

The results regarding the effect of soil characteristics on WF were not included in this paper due to the CROPWAT's inability to provide conclusive results regarding the relation between the soil type and the WF. However, future WF studies must further incorporate the influence of different soil types on their results. Creating geographical “climatic regions” can help further increase the accuracy of the results. Another important geographic parameter that was taken into account is the drainage basin in which each field is located. The availability of high spatial resolution is important for the full utilization of an integrated GIS-WF approach.

#### Modeling changes in crop distribution and agricultural practices

An additional benefit of the combined GIS-WF approach is the enhanced possibility for modeling the impact of changes in spatial crop distribution and specific agricultural practices on the WF. For example, one can easily model the impact of changing the types of crop grown or the influence of changing fertilization or cultivation practices on the WF. One example, analyzed in the present study, is the utilization of net-houses in all open-field banana fields. This change resulted in a decrease of 1.3 MCM year^−1^ of WF_regional.blue_ (Figure 10). In addition, changes to fertilization were also examined, showing a reduction of about. 3.2 MCM year^−1^ WF_regional.gray_ if 10% less fertilizer is applied. Eighty-seven percent of the reduction was in the SGB, suggesting that fertilization reduction efforts should be focused to this area.

In essence, using GIS allows to easily calculate the WF not only of the current situation, but how different scenarios can affect the WF_regional_. These scenarios can include adopting certain cultivation practices (such as soil-covering, net-house, compost vs. chemical fertilization and biological pesticides vs. chemical pesticides) and changing fertilization practices, as the examples in this study shows. In addition, different cropping systems, irrigation practices can be analyzed to show their impact on local and regional blue, green and gray WF. A combination multi-parameter changes can also be applied, in order to provide a more complex scenario. By integrating the energetic WF, and in the future additional parameters such as the financial WF, a wider picture of the energetic, financial and water-saving benefits of utilizing and adopting different agricultural practices and cropping system can be easily calculated for a certain area taking into account both the change in water-use and increase or decrease of crop yield.

#### Drainage basin driven analysis

The impact of the drainage-basin on the results (see Figure 9), reinforce the importance of the need for management and study of water resources at the spatial resolution of drainage basin of a specific water resource—lake, river, stream, or aquifer (Jaspers, 2003; Hoekstra et al., 2011). Today, many GIS software packages allow to compile drainage basin borders based on topographic data, which is readily available in many regions. An integrated GIS-WF basin-wide approach can help estimate the impact of different uses on the water availability in the basin. For agriculture, this approach can calculate the WF of specific crops or growing practices to water consumption and pollution in the entire catchment basin and model the effect of changes in crop distribution and agricultural practices on the total water availability. Comparing actual water consumption with the seasonal or yearly water availability in the basin with the combination of important approaches such as those outlined in UNEP's Integrated Water Resources Management can help facilitate and encourage a sustainable management of the region's water resources and rehabilitation of damaged aquatic ecosystems and water resources (Hassing et al., 2009).

## Conclusions

Using multiple WF parameters for comparing different crops revealed that classic WF yield parameter (m^3^ ton^−1^) alone is beneficial only when comparing identical or similar crops and is insufficient for comparison of different crops.

Additionally, the study emphasized the importance of determining the drainage basin of the study area and the strong impact of the environmental water quality standards used for the GWF calculation. The effect of these standards is especially significant where sensitive water bodies are involved as in the case of the SGB, where the WF_regional.gray_ constitutes the largest component of the WF_regional.total_ and is significantly higher than WF_gray_ results in the LJRB.

Finally, the research explored the benefits of utilizing high-resolution data with the help of GIS technology to study the effects of spatial factors (such as drainage basins, soil type, and climate areas) on the WF. Moreover, temporal changes (such as change in climate and precipitation over the years) can be used to observe and track changes in the WF. These high-resolution studies can provide data to help inform policy makers and farmers, and promote the planting of climate and soil appropriate crops and cultivation practices that lower the WF by increasing WUE and reducing the negative impact on the environment.

## Author contributions

All authors were involved in development of the project and analysis of the results. EST conducted the WF and GIS analysis.

### Conflict of interest statement

The authors declare that the research was conducted in the absence of any commercial or financial relationships that could be construed as a potential conflict of interest.
